# Genetic diversity, seasonality and transmission network of human metapneumovirus: identification of a unique sub-lineage of the fusion and attachment genes

**DOI:** 10.1038/srep27730

**Published:** 2016-06-09

**Authors:** Wei Zhen Chow, Yoke Fun Chan, Xiang Yong Oong, Liang Jie Ng, Siti Sarah Nor’E, Kim Tien Ng, Kok Gan Chan, Nik Sherina Hanafi, Yong Kek Pang, Adeeba Kamarulzaman, Kok Keng Tee

**Affiliations:** 1 Department of Medicine, Faculty of Medicine, University of Malaya, Kuala Lumpur, Malaysia; 2 Department of Medical Microbiology, Faculty of Medicine, University of Malaya, Kuala Lumpur, Malaysia; 3 Faculty of Information Science & Technology, Multimedia University, Melaka, Malaysia; 4 Tropical Infectious Diseases Research & Education Centre (TIDREC), Deputy Vice Chancellor (Research & Innovation), University of Malaya, Kuala Lumpur, Malaysia; 5 Division of Genetics and Molecular Biology, Institute of Biological Sciences, Faculty of Science, University of Malaya, Kuala Lumpur, Malaysia; 6 Department of Primary Care Medicine, Faculty of Medicine, University of Malaya, Kuala Lumpur, Malaysia

## Abstract

Human metapneumovirus (HMPV) is an important viral respiratory pathogen worldwide. Current knowledge regarding the genetic diversity, seasonality and transmission dynamics of HMPV among adults and children living in tropical climate remains limited. HMPV prevailed at 2.2% (n = 86/3,935) among individuals presented with acute respiratory tract infections in Kuala Lumpur, Malaysia between 2012 and 2014. Seasonal peaks were observed during the northeast monsoon season (November–April) and correlated with higher relative humidity and number of rainy days (*P* < 0.05). Phylogenetic analysis of the fusion and attachment genes identified the co-circulation of three known HMPV sub-lineages, A2b and B1 (30.2% each, 26/86) and B2 (20.9%, 18/86), with genotype shift from sub-lineage B1 to A2b observed in 2013. Interestingly, a previously unrecognized sub-lineage of A2 was identified in 18.6% (16/86) of the population. Using a custom script for network construction based on the TN93 pairwise genetic distance, we identified up to nine HMPV transmission clusters circulating as multiple sub-epidemics. Although no apparent major outbreak was observed, the increased frequency of transmission clusters (dyads) during seasonal peaks suggests the potential roles of transmission clusters in driving the spread of HMPV. Our findings provide essential information for therapeutic research, prevention strategies, and disease outbreak monitoring of HMPV.

Human metapneumovirus (HMPV) is a single-stranded RNA virus classified in the *Metapneumovirus* genus within the *Pneumovirinae* sub-family of *Paramyxoviridae*. Since the first description of HMPV among children in the Netherlands^1^, the virus has emerged as one of the main causative agents of acute respiratory tract infections (RTIs) across all ages worldwide, in addition to the human respiratory syncytial virus (HRSV)^2^,^3^,^4^,^5^,^6^,^7^,^8^. The clinical presentation of acute and severe RTIs due to HMPV are strikingly similar to that of HRSV which are mainly characterised by mild respiratory illnesses to severe cough, bronchiolitis, pneumonia and often accompanied by high fever, myalgia and vomiting^1^. In the United States for example, the annual disease burden due to HMPV in hospitalized children below 5 years of age has been estimated at 1 per 1000 children, similar to that of the influenza virus^9^.

HMPV is classified into two main genetic lineages denoted as genotypes A and B based on the phylogenetic analysis of the fusion (F) and attachment (G) genes. Each genotype is further classified into at least two sub-lineages – A1, A2, B1 and B2^10^. It was later reported that two additional genetic sub-lineages were described within the A2 sub-lineage, named A2a and A2b based on the phylogenetic analysis of the nucleocapsid (N) and F gene sequences in a population of paediatric patients^11^. Different genetic sub-lineages of HMPV also co-circulate in a temporal fashion and a shift in the predominant circulating strain is not uncommon^12^,^13^,^14^.

Acute respiratory infections caused by viruses such as HRSV, influenza A virus and human parainfluenza virus, amongst others are seasonally-distributed^15^. In temperate countries, a higher prevalence of HMPV has been reported during the months of winter and spring, with a lower seasonality than HRSV^2^,^13^,^14^,^15^,^16^. Climate also plays an important role in determining the seasonality of HMPV in tropical countries. Previous studies have described a direct correlation between the number of rainy days and the increase in the number of HMPV infections^6^,^8^. In addition to the number of rainy days, the effects of other important factors in a tropical climate such as the relative humidity, temperature and amount of rainfall on the prevalence of HMPV need to be investigated in order to provide a better understanding of its seasonality.

Molecular epidemiological surveillance can be used to infer the dynamics of disease transmission based on the genetic relatedness of viral sequences derived from the population, as shown for human immunodeficiency virus (HIV)^17^,^18^,^19^, influenza virus^20^,^21^, echovirus^22^ and ebola virus^23^, amongst others. Although HMPV is one of the main causative agents of RTIs worldwide, little is known about the temporal dynamics of HMPV transmission in the population. Transmission networks inferred from the analysis of viral sequences elucidate the degree of the spread of an infection in a subset of the population that allows the identification of the population at high risk of transmitting the virus amongst each other, thus enabling the implementation of effective and targeted public health preventive measures to reduce the prevalence of new infections in the population at large^17^,^18^,^24^.

To date, studies on the epidemiological distribution and genetic diversity of HMPV have been reported mainly among the hospitalised and outpatient children worldwide^4^,^7^,^12^,^25^,^26^. Despite the significant burden of acute RTIs due to HMPV^9^, information regarding the seasonal distribution, circulating genotypes and the dynamics of disease transmission of HMPV in the general population of all ages in tropical countries are currently limited^6^,^8^,^27^. In this study, we aimed to investigate the genetic diversity, seasonality and transmission network of HMPV infections among outpatient adults and children in Kuala Lumpur, Malaysia during a two-year period.

## Results

### Characteristics of the study population and seasonality of HMPV

**Figure 1:**
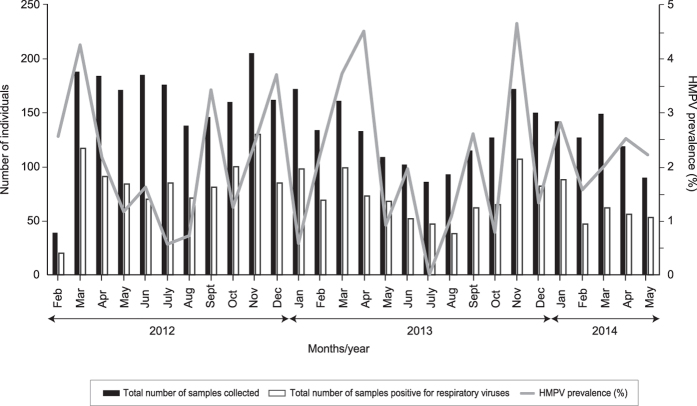
The distribution of human metapneumovirus (HMPV) among 86 adults and children presented with acute upper respiratory tract infections (URTI) between February 2012 and May 2014 in Kuala Lumpur, Malaysia. The monthly distribution of HMPV and its prevalence (number of HMPV-positive samples/total number of samples collected within the month × 100%) among a total of 3,935 nasopharyngeal samples.

Between February 2012 and May 2014, a total of 3,935 nasopharyngeal samples were collected (comprising 3,706 adults and 229 children), among which 86 (2.2%) samples were tested positive for HMPV. Among the infected individuals, seven (8.1%) were co-infected with other respiratory virus, whereby six individuals were dually co-infected with either rhinovirus (n = 3), adenovirus (n = 1), influenza B (n = 1) or coronavirus (n = 1) while one individual was triply co-infected with rhinovirus and influenza A. Co-infection with HRSV was not observed. The study population was comprised predominantly of females (61.6%, 53/86). Among 86 individuals infected with HMPV, 76.7% (n = 66) were adults (median age: 34.0 years, interquartile range, IQR, 26.0–54.0 years), 18.6% (n = 16) were elderly, defined as ≥65 years old^2^ (median age: 72.0 years, IQR, 69.8–75.0 years) and the remaining 4.7% (n = 4) were children (median age: 14.5 years, IQR, 12.8–16.3 years). The population was ethnically diverse, comprising of Malays (46.5%, 40/86), Indians (26.7%, 23/86), Chinese (23.3%, 20/86) and other ethnic groups (3.5%, 3/86). Overall, the patients reported a median total of 4.0 days (IQR, 3.0–7.0 days) since the onset of disease symptoms. Majority of the patients reported symptoms of cough (98.8%), followed by hoarseness of voice (89.5%), nasal discharge (72.1%), sneezing (70.9%), headache (69.8%), sore throat (69.8%), nasal congestion (67.4%) and muscle ache (65.1%). During the 28-months period, a higher prevalence of HMPV infections were reported in March 2012, April and November 2013 (Fig. 1).

Next, we assessed the effects of various meteorological factors in the context of a tropical climate on the prevalence of HMPV throughout the study period. The prevalence of HMPV significantly correlated with higher relative humidity (r = 0.449, *P* = 0.019) and number of rainy days (r = 0.440, *P* = 0.022). On the other hand, an inverse correlation between the prevalence of HMPV with the ground temperature (r = −0.281, *P* = 0.155) and particulate matter measuring 10 μm or less in diameter (PM10) (r = −0.163, *P* = 0.417) was observed, although the observation was statistically non-significant. The total amount of rainfall (r = 0.235, *P* = 0.258) was also associated with the prevalence of HMPV, although the observation was statistically non-significant.

### Phylogenetic analysis and genetic diversity of the F and G genes

**Figure 2:**
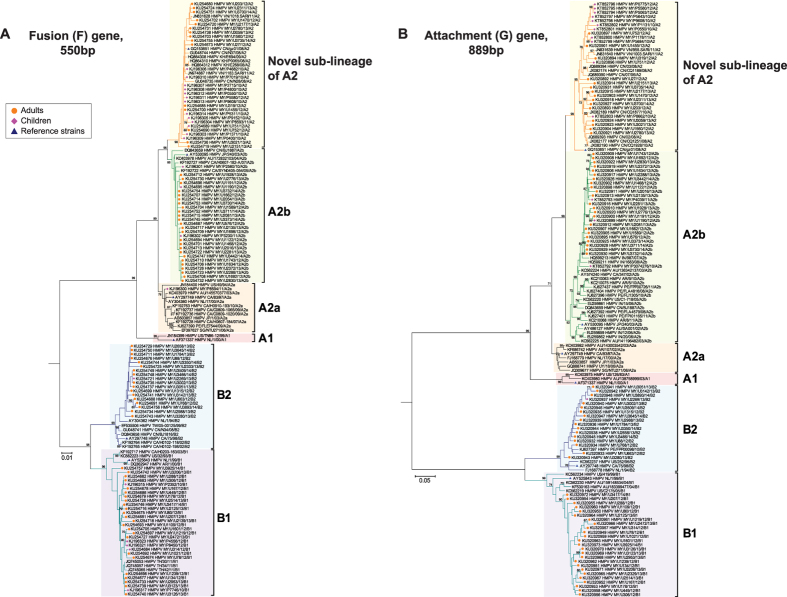
Phylogenetic analyses of the human metapneumovirus (HMPV) among the adults and children between February 2012 and May 2014 in Kuala Lumpur. The newly-sequenced (**A**) fusion (F) and (**B**) attachment (G) gene sequences from adults and children plus previously reported paediatric sequences from Malaysia^7^ were included in the neighbour joining tree analysis implemented in MEGA. Reference strains (blue triangles) of HMPV sub-lineages included AF371337 HMPV NL/1/00/A1, AY297749 HMPV CA/83/97/A2a, AY530095 HMPV JP/240/03/A2b, GQ153651 HMPV CN/gz01/08/A2, AY525843 HMPV NL/1/99/B1 and GQ153651 HMPV CA/75/98/B2^1^,^12^. All sequences were labelled according to the approved nomenclature of HMPV by the International Committee on Taxonomy of Viruses (ICTV)^53^. Country of origin for the reference and other database sequences was indicated in the tree by their respective country codes (AU, Australia; AR, Argentina; CA, Canada; KH, Cambodia, CN, China; IN, India; JP, Japan; NL, Netherlands; PE, Peru; SG, Singapore; TH, Thailand; TW, Taiwan; US, United States of America; UY, Uruguay and VN, Vietnam). The genotype classification was concordant between both F and G genes. Bootstrap values of greater than 70% were indicated on the branch nodes. The scale bars represent 1% and 5% genetic distances for F and G genes, respectively (0.01 and 0.05 substitutions per site).

**Figure 3:**
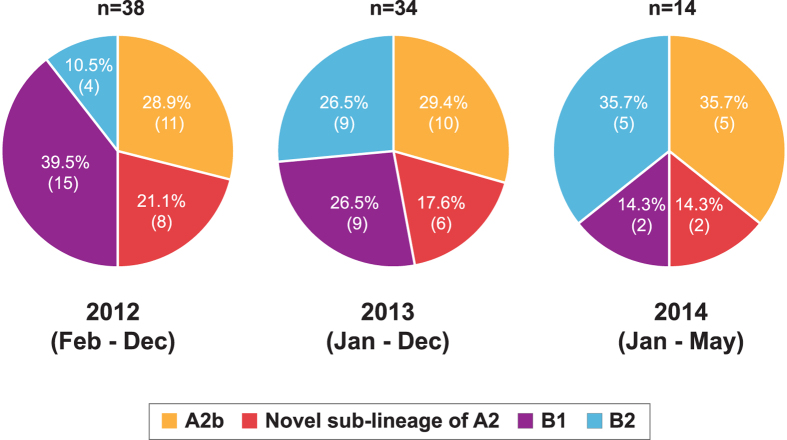
Annual prevalence of human metapneumovirus (HMPV) genotypes and sub-lineages co-circulating among 86 adults and children between 2012 and 2014 in Kuala Lumpur, Malaysia. The prevalence is denoted in % (n, frequency of each sub-lineage within that year).

Among the 86 samples positive for HMPV infection, the partial F and G genes were successfully amplified in 98.8% (85/86) and 95.3% (82/86) of the total samples, respectively. Phylogenetic analysis of the newly-sequenced and reference partial F and G gene sequences showed the clustering of the sequences into two main genotypes - A and B, and at least five known sub-lineages – A1, A2a, A2b, B1 and B2, in agreement with previous studies (Fig. 2A,B)^10^,^11^,^12^. Within genotype A, a total of 30.2% (26/86) of the newly-generated sequences clustered within the A2b sub-lineage. Interestingly, 18.6% (16/86) of the sequences clustered with a reference strain, HMPV CN/gz01/08/A2 (accession number: GQ153651) within a previously unrecognized sub-lineage of A2, supported by a strong bootstrap value of 98–99% in the phylogenies of both F and G genes. The unique clade was provisionally assigned as a novel sub-lineage of A2. In addition to Malaysia, previously published sequences from other countries (China, Vietnam, and Cambodia) were also grouped within this clade. Both A1 and A2a sub-lineages were not detected in our study population. Similarly, within genotype B, a total of 30.2% (26/86) of the sequences clustered within the B1 sub-lineage and 20.9% (18/86) of the sequences clustered within the B2 sub-lineage. In 2012, B1 sub-lineage circulated predominantly at 39.5% (15/38) of the population and a shift in the predominant genotype towards A2b (29.4%, 10/34) was observed in 2013 (Fig. 3). Between January and May 2014, both A2b and B2 sub-lineages co-circulated at equal prevalence at 35.7% (5/14). In addition to samples collected from four children in our current study population, phylogenetic analysis of the F and G genes sequenced from 21 paediatric patients recruited between April 2010 and December 2012 (median age: 1.84 years, IQR, 1.11–3.40) revealed that 52.0% (13/25) of the sequences clustered within the novel sub-lineage of A2, followed by A2b and B1 at 20.0% (5/25) each, and A2a and B2 at 4.0% (1/25) each (Fig. 2A,B). No sequences were detected in the A1 sub-lineage.

Based on the estimation of intra-genotype mean genetic distances of nucleotide and amino acid sequences of HMPV, F gene has higher sequence identities overall compared to G gene at both nucleotide (nt) and amino acid (aa) levels (F gene, nt: 88.7% and aa: 75.9% vs G gene: nt: 54.3% and aa: 27.6%, respectively) (Table 1). Sequences within the novel sub-lineage of A2 were highly identical at both nucleotide and amino acid levels in both F and G genes (F gene, nt: 99.1% and aa: 97.5% and G gene: nt: 95.7% and aa: 95.5%). Next, we estimated the inter-genotype mean genetic distances of the HMPV genotypes and sub-lineages (Table 2). Sub-lineage distances between genotypes A and B were high in both F and G genes, estimated around 17.7–19.3% and 77.8–88.3%, respectively. Sub-lineage distances within genotype were estimated around 5.9–7.5% and 22.6–26.9% in the F and G genes, respectively. Within A2, genetic distances between sub-lineages A2a and A2b were 3.8% in the F gene and 13.0% in the G gene. Notably, the genetic distances between these sub-lineages and the novel sub-lineage of A2 were similar or higher, estimated around 3.6–4.4% and 11.9–12.9% in both the F and G genes, respectively. Specific amino acid substitutions were also present within the novel sub-lineage of A2 in the G protein, but not in the F gene. In comparison with A2a and A2b sub-lineages, eleven specific amino acid substitutions in the novel sub-lineage of A2 were identified at positions 40, 59, 93, 106, 148, 164, 188, 197, 199, 216 and 227 (Table 3). Taken together, based on the phylogenetic tree topology, genetic distances and specific amino acid substitution analyses in both the F and G genes, the newly identified sub-lineage was distinct from the previously described A2a and A2b sub-lineages.

### HMPV transmission network

**Figure 4:**
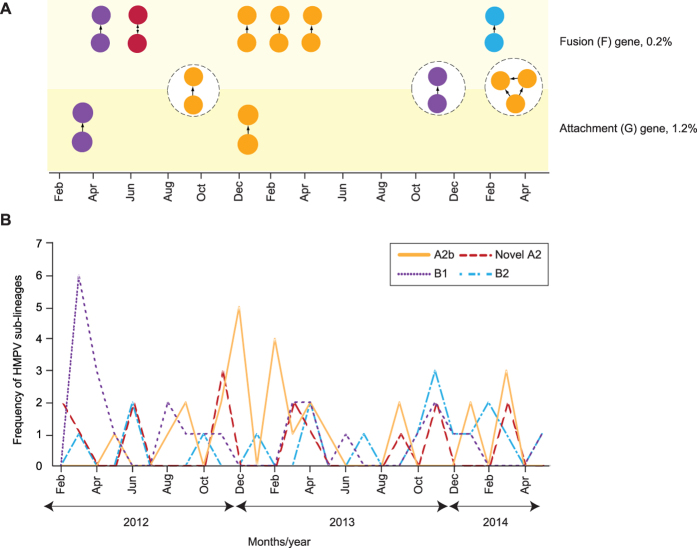
Human metapneumovirus (HMPV) transmission network among the adults and children in Kuala Lumpur between February 2012 and May 2014. The transmission network was inferred from the newly-sequenced F (n = 85) and G (n = 82) genes based on the Tamura-Nei 93 (TN93) pairwise distance estimates^51^ performed using a custom script in Python (release 3.2.6) with 1,000 bootstrap replicates. (**A**) The monthly distribution of transmission clusters inferred for F and G genes at the genetic distance cutoffs of 0.2% and 1.2%, respectively. The three common transmission clusters identified in both genes were highlighted in circles (dotted lines). Probable direction of disease transmission within a cluster was estimated by placing a directed edge from the putative “donor” node to the “recipient” node(s), where the estimated date of infection, EDI_donor_ is older than the EDI_recipient_^18^. (**B**) The monthly frequency (in absolute numbers of samples/individuals) of HMPV sub-lineages co-circulating in the study population. HMPV sub-lineages A2b, B1, B2, and the novel sub-lineage of A2 were indicated by their respective colours.

In order to elucidate the dynamics of HMPV transmission among the adults and children population in the present study, we constructed transmission networks based on the pairwise distances using the TN93 model estimated from the newly-sequenced F and G genes. The lower 0.025 percentile of the inter-person genetic distances from database sequences, which represented the patristic distance cutoff for transmission cluster interpretation, were estimated at 0.2% and 1.2% for the F and G genes, respectively (Supplementary Table 1). In the F gene data set, 19 sequences (22.4%, 19/85) formed a total of nine clusters (comprising of eight dyads and one network) while in the G gene, 11 sequences (13.4%, 11/82) formed a total of five clusters (comprising of four dyads and one network), ranging between two and three individuals in size (Fig. 4A). Of these clusters, only two dyads and one network were concordant in both genes. Overall, more transmission clusters were observed in the A2b sub-lineage than other sub-lineages in both the F (57.9%, 11/19) and G (63.6%, 7/11) genes. A known transmission pair of two epidemiologically-linked adult and child infected with the novel A2 sub-lineage was also observed in the F (but not in the G) gene. HMPV has been reported to be highly infectious within the first 4–6 days of infection^28^, after which its ability to cause infection in another individual declines. Hence we proceed to estimate the difference between estimated date of infection (denoted as ΔEDI) between existing members of clusters observed in the F and G genes to evaluate the reliability of the genetic-based approach in inferring transmission network. We found that a majority of the clusters inferred in the F (8/9 clusters) and G genes (4/5 clusters) had ΔEDIs of ≤7 days which provided substantial validation of the method used in this study (Fig. 4A). Of note, the frequency of transmission clusters identified during the study period coincided with an increase in the detection of the respective HMPV sub-lineages in the population, as observed in the A2b (between September 2012 and April 2013) and B1 (between March and April 2012) sub-lineages (Fig. 4A,B).

## Discussion

To date, most studies were focused on describing the molecular epidemiology of HMPV in hospitalised children^4^,^7^,^12^,^25^,^26^ and consequently, knowledge regarding the prevalence and genotypic distribution among the general population of all ages are limited^6^,^29^. In addition, the seasonality and dynamics of disease transmission in countries with a tropical climate have not been analysed in detail. In the present study, we reported the recent molecular epidemiology of HMPV in an outpatient population of adults and children presented with symptoms of acute URTI in Kuala Lumpur during a two-year period. From 3,935 nasopharyngeal swabs collected between 2012 and 2014, the average prevalence of HMPV in the population was fairly low at around 2%, similar to previous studies conducted across all ages^6^. Conversely, HMPV was more prevalent among children in the hospitalised or community setting, with a reported prevalence of over 5% particularly among the young and immunocompromised children^1^,^3^,^4^,^25^,^30^. Although the overall prevalence of HMPV infection was low in our study population, a relatively higher monthly prevalence of up to 4–5% was observed during the November–April period (Fig. 1), coincided with the northeast monsoon season which typically occurs in the region between October and March. We also showed that the prevalence of HMPV was significantly correlated with high relative humidity and the number of rainy days.

Throughout the study period, both HMPV genotypes A and B, including three known sub-lineages – A2b, B1 and B2 co-circulated all year round. Meanwhile, sub-lineages A1 and A2a were not detected in our study population. Similarly, sub-lineage A1 which circulated in China^31^, was also not detected in the neighbouring regions including Cambodia^6^, Thailand^8^ and Singapore^4^; and worldwide after 2006, in Austria^13^, Argentina^32^, Australia^33^, Canada^12^, and the United States^34^,^35^. On the other hand, sub-lineage A2a circulated widely at a low prevalence in other countries including Thailand^8^ and Canada^36^. Both A2b and B1 circulated predominantly in the population, with a combined prevalence of around 60% (Fig. 3). We also observed a shift in the predominant circulating genotype from sub-lineage B1 to A2b in the population between 2012 and 2013, although a longer study period with more samples may be required to provide an accurate representation. Recently, genotype A2 has been associated with a more severe disease in hospitalised children in Jordan and Cambodia^6^,^37^. Further studies to assess the impact of HMPV genotypes on disease severity are warranted. In addition to the known sub-lineages, a previously unreported sub-lineage of A2 was identified at a lower prevalence at around 19% in the population. Phylogenetic and pairwise distance analyses of the F and G genes sequence data including all publicly available global reference sequences confirmed the novelty of the sub-lineage which was distinct from other known sub-lineages within A2, such as A2a and A2b (Fig. 2, Table 2). More than half of the F and G sequences previously amplified among Malaysian children between 2010 and December 2012^7^ were grouped within the novel sub-lineage of A2. Furthermore, several reference F and G gene sequences isolated from neighbouring countries including Vietnam, Cambodia and China, sampled between 2006 and 2011 also clustered with the Malaysian sequences within the novel sub-lineage of A2 and further suggesting that the novel sub-lineage of A2 might have been circulating in the region at a lower prevalence earlier (Fig. 2). Further studies however are required to estimate its time of emergence in our study population and whether the proposed novel sub-lineage of A2 is also circulating in other regions besides Southeast and East Asia. Complete genome sequencing followed by rigorous phylogenetic and genetic distance analyses of the novel sub-lineage of A2 are necessary to confirm its assignment as a distinct sub-lineage.

Analysis of epidemiological data is generally useful to estimate the temporal prevalence and seasonality of a viral pathogen. However the inclusion of transmission network information could elucidate the spread and dynamics of a disease attributed to specific genotypes or sub-lineages circulating in the population that further corroborate the observation from epidemiological surveillance of a disease. Using programming language-based approach, we inferred the transmission network of HMPV in the population based on the genetic sequences of the F and G genes. To the best of our knowledge, such analysis represents the first of which the spread and dynamics of HMPV transmission in the general population was mapped. Using the estimated cutoffs for the pairwise genetic distance between HMPV sequences and the EDI between individuals, below which individuals were classified as a transmission cluster, we identified up to nine transmission clusters (in the F gene) of HMPV circulating in the cohort between 2012 and 2014 (Fig. 4A). The size of these clusters was generally small (dyads) and involved various HMPV sub-lineages, suggesting the presence of multiple sub-epidemics in the population with no apparent evidence of a major outbreak. Nonetheless, the increased frequency of transmission clusters correlated with the incidence of HMPV detection (Fig. 3B), indicating the potential roles of transmission clusters in driving the spread of respiratory infections. We were also able to estimate the directionality of viral transmission between individuals within the cluster, which allows the identification of the putative donor/recipient that may be important in facilitating efforts to minimize forward transmission^18^. Nevertheless, it is important to note that the transmission clusters inferred herein reflected the genetic relatedness of the viral sequences and their direction of transmission, without implying an actual (direct) viral transmission between clustered individuals^18^,^19^,^24^.

In order to understand the potential transmission and spread of infectious diseases such as the pandemic influenza, there has been a considerable amount of research on the role of social contact network in spreading a disease within the community. These studies used conceptualized frameworks^38^ or computational models^39^ to investigate the social contact network among schoolchildren and teenagers, and simulate how network interventions can significantly contain the spread of influenza within a community. However, most studies relied on the use of survey and contact diaries method which are laborious and often unreliable due to self-report inaccuracies, such as incomplete or illegible survey forms^38^ and failure to recall their network members precisely^40^. Therefore, our method presents a simple and unbiased approach to construct the transmission network based on the clustering of genetically-related viral strains sequenced from the infected individuals.

Although the potential use of transmission cluster in understanding HMPV spread has been demonstrated here, limitation does exist in the analysis, such as the lack of vital epidemiological information and the observed discordant clustering generated by different genetic regions. Firstly, to improve the cluster resolution and minimize potential discrepancies in the analysis, estimated clusters should be interpreted in parallel with information of contact history among members of a cluster, if available^41^. Likewise, the suitability of a genetic region can be determined through known transmission pairs sampled from well-documented outbreaks by assessing the reproducibility of the cluster features using multiple partial and even the complete genomes. In addition, although cluster overestimation may be unlikely in our analysis where the upper distance cutoffs were set below the lower 0.025 percentile of inter-person genetic distances^24^,^42^, the availability of the intra-person genetic distances will nonetheless provide a better clue on the lower distance cutoffs. Due to the interim and self-limiting nature of the infection, it is indeed a challenge to collect serial samples throughout acute illness, which will not immediately benefit the patients. Lastly, as in many other molecular epidemiological surveillance studies, only a subset of the population was recruited and analysed. Therefore, the size, frequency and structure of the transmission clusters observed could have been more accurate by increasing the sample size involving more recruitment sites (but not necessarily longer study duration).

In summary, the present study characterised the recent prevalence, seasonality and genetic diversity of HMPV in a large outpatient population presented with acute respiratory tract infections in Kuala Lumpur, Malaysia. The incorporation of transmission network analysis further demonstrated a deeper understanding of HMPV transmission dynamics in the population. Due to the epidemiological impact of HMPV in causing acute respiratory infections, continuous molecular surveillance is warranted to inform the development of therapeutic and preventive approaches, and to facilitate the monitoring of disease outbreaks in the population.

## Materials and Methods

### Ethics statement

The study was approved by the University Malaya Medical Centre (UMMC) Medical Ethics Committee. Standard, multilingual consent forms allowed by the Medical Ethics Committee were used and written consent was obtained from all study participants. All experiments were performed in accordance with approved guidelines and regulations.

### Study population and sample collection

Nasopharyngeal swabs were collected from adults and children (aged 18 years and below) attending the primary care clinic in UMMC, Kuala Lumpur, Malaysia for a period of 28 months between February 2012 and May 2014. Eligible individuals were those who experienced symptoms of acute upper RTIs (URTI) for not more than two weeks at the time of study recruitment, defined as the presence of either sneezing, nasal discharge/congestion, headache, sore throat, hoarseness of voice, muscle ache and cough, as implemented in other studies on both naturally acquired and experimental colds^43^,^44^,^45^,^46^. The estimated number of days since the development of first disease symptom (estimated days after disease onset) was recorded. Nasopharyngeal swabs were stored in universal transport medium (Copan Diagnostics, California, USA) and transported to the laboratory for immediate processing.

### Detection of human metapneumovirus

Total viral nucleic acid was extracted from 200 μL of specimen using the NucliSENS easyMAG automated platform (bioMerieux, Durham, North Carolina, USA) according to the manufacturer’s recommendation. One-step multiplex PCR was performed using the xTAG Respiratory Viral Panel (RVP) FAST Assay (Luminex Molecular Diagnostics Inc., Toronto, Canada) according to the manufacturer’s recommendation^47^. An internal positive control (*Escherichia coli* phage MS2) and a positive run control (bacteriophage lambda DNA) were included to monitor the assay performance. The resulting median fluorescence intensities were analysed using the Luminex 200 IS platform (Luminex Molecular Diagnostics Inc.).

### Amplification and sequencing of the F and G genes

Viral RNA from HMPV-positive samples was reverse-transcribed to cDNA using SuperScript III RNase H^−^ Reverse Transcriptase (Invitrogen, Carlsbad, California, USA) and random hexamers (Applied Biosystems, Foster City, California, USA). The partial F open reading frame (ORF) (550 bp) and the complete G ORF (580–889 bp) of HMPV were amplified using primers as described previously^10^ using BIO-X-ACT Short DNA Polymerase enzyme (Bioline Reagents Ltd, UK) under the following thermocycling conditions: initial denaturation at 95 °C for 5 minutes, followed by 35 cycles of denaturation at 94 °C for 30 seconds, annealing at 55 °C for 1 minute and elongation at 72 °C for 1 minute, and a final elongation step at 72 °C for 10 minutes. Overall, two separate sets of nested primers were used to amplify the G genes. For specimens that failed to be amplified, we used the following sets of newly designed nested primers: outer primers set F1_6206, 5′-AAAACAARAAWATGGGACAAG-3′ and R1_7176, 5′-TCAGGRAGATARACATTRACAG-3′; and inner primers set F2_6258, 5′-RGCRAYWGACATGYTCAAAG-3′ and R3_7139, 5′-GATCCATTGYYATTTRTCYC-3′. Population sequencing was performed in both directions using an ABI PRISM 3730XL DNA Analyzer with BigDye terminators (Applied Biosystems). The newly-generated F and G gene sequences in this study were submitted in GenBank (accession numbers: KU254673 to KU254757 and KU320892 to KU320973).

### Phylogenetic and sequence analysis

Global (n = 214) and regional (n = 170) published nucleotide sequences of HMPV F and G genes were retrieved from the GenBank database and aligned with the newly-generated sequences using the PRANK algorithm available on the GUIDANCE server^48^,^49^. In addition, 21 HMPV F and G sequences amplified among paediatrics diagnosed with acute lower RTIs by our laboratory were also included in the phylogenetic analysis (GenBank accession numbers: KJ196300 to KJ1963023 and KT852792 to KT852803). These samples were collected between April 2010 and December 2012 and were laboratory-confirmed as HMPV-positive by direct fluorescent-antibody staining and/or virus culture, as described previously^7^. Reference strains of HMPV genotype A and B sub-lineages comprising AF371337 HMPV NL/1/00/A1, AY297749 HMPV CA/83/97/A2a, AY530095 HMPV JP/240/03/A2b, GQ153651 HMPV CN/gz01/08/A2, AY525843 HMPV NL/1/99/B1 and GQ153651 HMPV CA/75/98/B2 were included^1^,^12^. Phylogenetic trees were constructed by the neighbour joining method based on the Kimura two-parameter model with a transition-transversion ratio of 2.0 implemented in MEGA 5.05^50^. The reliability and robustness of the branching orders were analysed by bootstrap analysis of 1,000 replicates. The mean genetic distances of nucleotide and amino acid sequences within and between genotypes/sub-lineage were estimated using the Kimura two-parameter model in MEGA.

### HMPV transmission network

The HMPV transmission network was inferred from the newly-sequenced F and G genes based on the Tamura-Nei 93 (TN93) pairwise distance estimates^51^ performed using a custom script in Python (release 3.2.6) with 1,000 bootstrap replicates. A transmission cluster is characterised by the presence of at least two individuals (represented by nodes) whose viral sequences are genetically linked (represented by edges) at a given genetic distance threshold or cutoff^18^,^19^. The genetic distance cutoff values can be determined between the highest and lowest values of intra- and inter-person distances (known as the patristic distances), respectively^24^,^42^. However, since HMPV is associated with acute infection, measuring intra-person viral genetic distance is often challenging and impractical due to rapid viral clearance within a host. We therefore estimated the most probable cutoff values by determining the lower 0.025 percentile of the inter-person genetic distances calculated from the global F (n = 66) and G (n = 45) reference sequences representing all known genotypes and sub-lineages. Sequences from different individuals with genetic distance falling below these cutoffs were classified as transmission cluster. Clusters were described as dyads if they contain two nodes, and network if >2 nodes^19^. The estimated date of infection (EDI) for each individual was predated from the number of days after disease symptoms onset as reported by the patients and the incubation period of 4–6 days for HMPV^28^. Difference between EDI for each individual was denoted as ΔEDI. In addition to genetic distance cutoff, adjacent nodes with a ΔEDI of less than 30 days were considered in cluster construction (viral shedding beyond 30 days following primary infection is probably uncommon, hence viral transmission may be very limited or absent). Such criterion was used for an acute infection in order to improve the analytical power of cluster determination by effectively eliminating genetically similar but epidemiologically unrelated viruses that may complicate transmission cluster interpretation. Probable direction of virus transmission within a cluster was also estimated by placing a directed edge from the putative “donor” node to the “recipient” node(s), where the EDI_donor_ is older than the EDI_recipient_^18^.

### Statistical analysis

We analysed the effects of various climatic factors, namely the daily ground temperature, relative humidity, number of rainy days, total amount of rainfall and PM10, on the prevalence of HMPV in Kuala Lumpur during the study period. All important meteorological parameters provided by the Malaysian Meteorological Department were obtained from a weather station (coordinates: 3°06′N (latitude) and 101°39′N (longitude)) situated approximately 2 km away from UMMC^52^. Data was analysed using Pearson correlation coefficient and performed on the SPSS statistical package (version 21.0, IBM).

## Additional Information

**How to cite this article**: Chow, W. Z. *et al*. Genetic diversity, seasonality and transmission network of human metapneumovirus: identification of a unique sub-lineage of the fusion and attachment genes. *Sci. Rep.* **6**, 27730; doi: 10.1038/srep27730 (2016).

## Supplementary Material

Supplementary Information

## Figures and Tables

**Table 1:** Estimated mean sequence identities of the human metapneumovirus (HMPV) genotype and sub-lineages in the fusion (F) and attachment (G) genes at the nucleotide and amino acid levels.

Gene/protein	Genotype/sub-lineage	Mean identity (%)	
		Nucleotide	Amino acid
**F**	**Overall**	**88.7**	**75.9**
	Within A	96.7	90.4
	Within A1	98.8	96.7
	Within A2a	99.0	97.7
	Within A2b	98.6	95.8
	Within novel A2 sub-lineage	99.1	97.5
	Within B	95.8	91.3
	Within B1	98.4	95.8
	Within B2	98.5	96.5
**G**	**Overall**	**54.3**	**27.6**
	Within A	88.6	81.9
	Within A1	93.9	90.5
	Within A2a	96.6	94.8
	Within A2b	95.0	93.2
	Within novel A2 sub-lineage	95.2	91.9
	Within B	84.3	74.2
	Within B1	96.5	94.7
	Within B2	95.6	91.1

**Table 2:** Estimated mean genetic distances between each genotype and sub-lineage of human metapneumovirus (HMPV) in the fusion (F) and attachment (G) genes (in %).

Gene	Genotype/sub-lineage	A1	A2a	A2b	Novel A2 sub-lineage	B1	B2
F	A1		0.9	1.1	1.2	1.8	1.9
	A2a	5.9		0.7	0.8	2.0	1.9
	A2b	7.5	3.8		0.7	2.1	2.0
	Novel A2 sub-lineage	7.9	4.4	3.6		2.0	2.0
	B1	17.7	18.4	19.0	18.7		1.1
	B2	18.2	18.5	19.3	19.3	7.0	
G	A1		1.7	1.8	2.0	5.4	4.7
	A2a	22.6		1.1	1.2	4.9	5.0
	A2b	25.0	13.0		1.0	5.4	5.6
	Novel A2 sub-lineage	27.4	12.9	11.9		5.5	6.0
	B1	87.7	78.5	85.7	85.8		2.1
	B2	81.8	77.8	86.6	88.3	26.9	

Standard error estimates of the mean genetic distances are shown in the upper diagonal panel.

**Table 3:** Lineage-specific amino acid substitutions within the human metapneumovirus (HMPV) sub-lineages of A2 in the attachment (G) protein open reading frame between position 14 and 228^a^.

A2 sub-lineage	Amino acid position in the G gene										
	40	59	93	106	148	164	188	197	199	216	227
A2a	T	T	H	P	V	P	T	T	T	T	P
A2b	T	T	H	P	V	P	T	T	V	T	P
Novel sub-lineage of A2	A	A	L	S	A	S	A	I	A	A	T

^a^The position of amino acid was determined according to HMPV reference genotype A2, AY304360 HMPV NL/17/00/A2a.
