# Supplementary material for: Genetic diversity, seasonality and transmission network of human metapneumovirus: identification of a unique sub-lineage of the fusion and attachment genes
Source: Sci Rep. 2016 Jun 9;6:27730. doi: 10.1038/srep27730 (PMC4899729; doi:10.1038/srep27730)
Supplement: Supplementary Information [file srep27730-s1.pdf]

Genetic diversity, seasonality and transmission network of human metapneumovirus: identification of a unique sub-lineage of the fusion and attachment genes

Authors:

Wei Zhen Chow, Yoke Fun Chan, Xiang Yong Oong, Liang Jie Ng, Siti Sarah Nor'E, Kim Tien Ng, Kok Gan Chan, Nik Sherina Hanafi, Yong Kek Pang, Adeeba Kamarulzaman, and Kok Keng Tee

Supplementary Table 1. Inter-person genetic distances and cutoff values estimated based on a set of human metapneumovirus (HMPV) reference sequences representing all known genotypes and sub-lineages.

**Gene**	**Genotype/sub-lineage (n)**	**Genetic distance[a]**		
		Lower 2.5% percentile (95% CI)	Lower 5.0% percentile (95% CI)	Selected cutoff values
**F**	A1 (6)	.002 (.002-.007)	.002 (.002-.007)	.002
	A2a (15)	.002 (.002-.003)	.002 (.002-.004)	
	A2b (11)	.003 (.002-.005)	.004 (.002-.007)	
	Novel sub-lineage of A2 (8)	.002 (.002-.002)	.002 (.002-.003)	
	B1 (12)	.003 (.002-.007)	.004 (.003-.010)	
	B2 (14)	.002 (.002-.004)	.004 (.002-.005)	
**G**	A1 (5)	.012 (.012-.046)	.012 (.012-.046)	.012
	A2a (16)	.014 (.002-.017)	.016 (.014-.023)	
	A2b (9)	.018 (.018-.029)	.018 (.018-.032)	
	Novel sub-lineage of A2 (5)	.021 (.021-.037)	.021 (.021-.037)	
	B1 (4)	.040 (.040-.059)	.040 (.040-.055)	
	B2 (6)	.028 (.028-.055)	.028 (.028-.055)	

[a] expressed in nucleotide substitutions per site.
